# Tinnitus Prevalence in the Adult Population—Results from the Gutenberg Health Study

**DOI:** 10.3390/medicina59030620

**Published:** 2023-03-20

**Authors:** Berit Hackenberg, Karoline O’Brien, Julia Döge, Karl J. Lackner, Manfred E. Beutel, Thomas Münzel, Norbert Pfeiffer, Andreas Schulz, Irene Schmidtmann, Philipp S. Wild, Christoph Matthias, Katharina Bahr-Hamm

**Affiliations:** 1 Department of Otorhinolaryngology, University Medical Center Mainz, 55131 Mainz, Germany; berit.hackenberg@unimedizin-mainz.de (B.H.);; 2 Institute for Clinical Chemistry and Laboratory Medicine, University Medical Center Mainz, 55131 Mainz, Germany; 3 Department of Psychosomatic Medicine and Psychotherapy, University Medical Center Mainz, 55131 Mainz, Germany; 4 Department of Cardiology—Cardiology I, University Medical Center Mainz, 55131 Mainz, Germany; 5 Department of Ophthalmology, University Medical Center Mainz, 55131 Mainz, Germany; 6 Preventive Cardiology and Preventive Medicine—Department of Cardiology, University Medical Center Mainz, 55131 Mainz, Germany; 7 Institute of Medical Biostatistics, Epidemiology and Informatics, University Medical Center Mainz, 55131 Mainz, Germany; 8 Center for Thrombosis and Hemostasis, University Medical Center Mainz, 55131 Mainz, Germany; 9 DZHK (German Center for Cardiovascular Research), Partner Site RhineMain, 60549 Mainz, Germany; 10 Institute of Molecular Biology (IMB), 55128 Mainz, Germany

**Keywords:** cohort study, hearing loss, audiology, otology/neurotology

## Abstract

*Background and Objectives*: Tinnitus is a common symptom in medical practice, although data on its prevalence vary. As the underlying pathophysiological mechanism is still not fully understood, hearing loss is thought to be an important risk factor for the occurrence of tinnitus. The aim of this study was to assess tinnitus prevalence in a large German cohort and to determine its dependence on hearing impairment. *Materials and Methods*: The Gutenberg Health Study (GHS) is a population-based cohort study and representative for the population of Mainz and its district. Participants were asked whether they suffer from tinnitus and how much they are burdened by it. Extensive audiological examinations using bone- and air-conduction were also performed. *Results*: 4942 participants (mean age: 61.0, 2550 men and 2392 women) were included in the study. The overall prevalence of tinnitus was 26.1%. Men were affected significantly more often than women. The prevalence of tinnitus increased with age, peaking at ages 75 to 79 years. Considering only annoying tinnitus, the prevalence was 9.8%. Logistic regression showed that participants with severe to complete hearing loss (>65 dB) were more likely to have tinnitus. *Conclusions*: Tinnitus is a common symptom, and given demographic changes, its prevalence is expected to increase.

## 1. Introduction

Tinnitus is a frequently reported symptom in otolaryngologic practice. It can be defined as a sound that is perceived without an external source [1]. Some people have difficulty localizing their tinnitus, while others attribute it to one of the two ears [1].

Although it is a common condition, there is still no standardized classification of tinnitus [2]. Patients may define their tinnitus by the limitations they experience in daily life, while others may not be bothered by their tinnitus [2]. Studies have reported highly variable tinnitus prevalence, ranging from 4% to 37% worldwide and from 9% to 29% in Europe [3,4,5,6]. Some authors also report tinnitus defined according to the time of its occurrence or according to the perceived distress [7,8,9,10].

Hearing impairment is considered one of the most important risk factors for tinnituss [1]. Although knowledge about the pathophysiological mechanisms is still sparse, it is assumed that hearing loss reduces nerval activity, which downregulates cortical inhibition. This in turn leads to a hyperexcitability in the auditory cortex [11]. In the auditory cortex, the overstimulation is assumed to be perceived as tinnitus [12,13]. This knowledge comes from animal models, but the translation of these pathomechanisms to the clinical phenomenon of tinnitus remains difficult [14,15]. For the imbalance in neural activity to be perceived as tinnitus, the primary sensory cortex must interact with other, higher-ordered networks such as the limbic system [16,17]. Jastreboff proposed that the negative experience of tinnitus stimulates the limbic system, which activates the sympathetic nervous system and leads thereby to a catecholamine released [17]. This strengthens the negative perception of tinnitus. Without this further processing, tinnitus might not trigger negative emotions and habituation might occur. This model is based on classical conditioning and although transferability from animal studies remains difficult, it is a widely accepted model in the clinical understanding of tinnitus [18].

The causal relationship between hearing loss and tinnitus is supported by the fact that patients with either unilateral or bilateral profound hearing loss and tinnitus experience a significant improvement in their tinnitus after being fitted with a cochlear implant to restore hearing [19,20]. This positive effect is also observed in patients with hearing aids, provided that the tinnitus is localized in the amplification range of the hearing aids (frequencies up to 6 kHz) [21,22].

Furthermore, despite this association of hearing impairment and tinnitus, few studies reporting on the prevalence of tinnitus have included hearing impairment tested for by pure-tone audiometry in their study design [23,24]. Larger cohort studies are sometimes conducted using a mailed questionnaire and therefore rely on the participants’ self-assessment [25]. Louw et al. could show that self-reporting of hearing loss may not sufficiently detect hearing impairment [26]. Therefore, it seems necessary to include pure-tone audiometry in the reporting of tinnitus prevalence. Once we understand the neurophysiological model of tinnitus as described above, we must assume that secondary emotional processing plays an important role in the development of tinnitus. This could be due to the cultural and linguistic differences between populations and might explain the highly scattered prevalence figures in different countries [5].

Given the constantly changing age distribution of the German population, tinnitus prevalence may vary over time. Therefore, comparing ongoing reports of tinnitus prevalence seems crucial to understanding how otolaryngologic disorders burden such a population. To date, no previous study has investigated the prevalence of tinnitus with reference to the hearing loss rate (as defined by pure-tone audiometry) in a large German cohort. The purpose of this study is to determine the prevalence of tinnitus in a large, population-based, randomly selected cohort, and to correlate the occurrence of tinnitus with hearing loss, as determined by pure-tone audiometry.

## 2. Materials and Methods

The Gutenberg Health Study (GHS) is a large, population-based cohort study. As a monocentric study, it covers the area of the city of Mainz and the district of Mainz-Bingen. By randomly drawing participants from the residents’ registration office, the study results are representative for this area. In addition, the study cohort is stratified by sex, residence (rural vs. urban) and age. The GHS was initiated in 2007 at the University Hospital Mainz, Germany. Ten years later, the first study phase (baseline) was successfully completed. At the 10-year follow-up after baseline (10-FU, from 2017 and 2020), an otologic examination was added to the study protocol. Participants were asked “Do you suffer from ringing in the ears (tinnitus)?” (yes/no). They were also asked, “How much do you feel burdened by it?” and requested to rate their tinnitus on a six-level scale ranging from “little stressful” (=1) to “extremely stressful” (=6). Pure-tone audiometry for air- and bone-conduction was performed at an otologic testing station by trained study nurses. Testing was performed separately for both ears at the following frequencies: 0.125, 0.25, 0.5, 0.75, 1, 2, 3, 4, 6, 8 and 10 kHz. All tests were performed with the Auritec^®^ AT1000 clinical audiometer and in a soundproof booth. A detailed description of the methodology can be found elsewhere [27,28].

Participants with missing data on tinnitus (yes/no) and/or missing data on pure-tone audiometry in the frequency 0.5, 1, 2 or 4 kHz were retrospectively excluded. Results were stratified by sex and age (in 5-year increments). Hearing impairment was classified according to the updated WHO classification of hearing impairment [29]. Results were weighted using the European Standard Population (ESP) of 2013 [30]. In addition, logistic regression analysis was performed to test whether there were factors that increased the likelihood of tinnitus. All statistical analyses were performed using R version 4.1.0 (2021-05-18). 

The study was approved by the local institutional review board (Ethics Commission of the State Chamber of Physicians of Rhineland-Palatine, reference no. 837.020.07) and was conducted in full compliance with the Declaration of Helsinki. Written informed consent was obtained from all subjects before participation in the study.

## 3. Results

**Figure 1:**
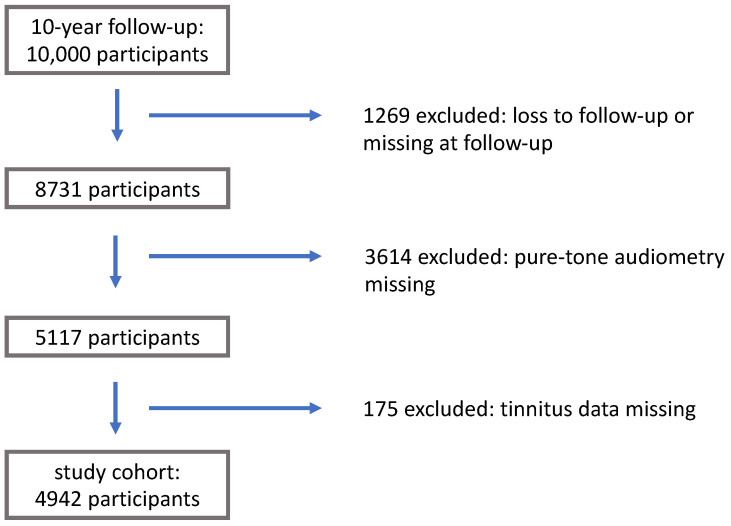
Flow-chart for patient selection.

The GHS cohort included 15,010 participants at baseline. This study was conducted after the first 10,000 participants were invited to attend the 10-FU. Figure 1 shows the loss to follow-up and exclusion process for the study cohort. 

**Figure 2:**
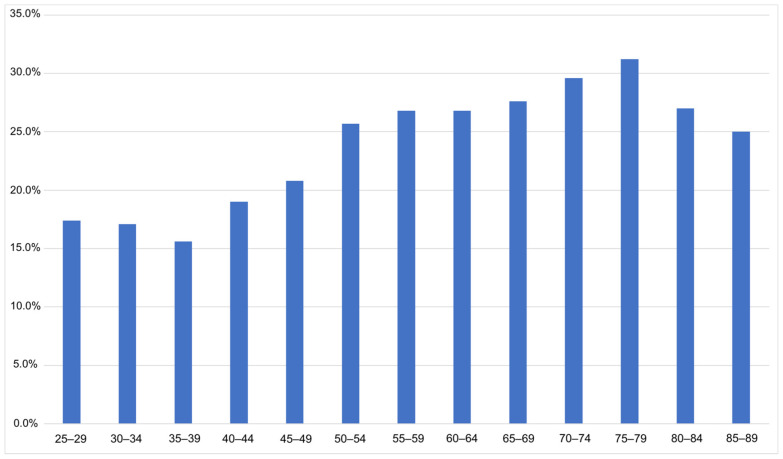
Prevalence of tinnitus by age group (*x*-axis: age groups in 5 year increments; *y*-axis: prevalence in %).

A total of 4942 participants were included (2550 men and 2392 women). The mean age was 61.0 years (SD: 13.3) (see Table 1). Of the 4942 participants, 1289 indicated that they had tinnitus (26.1%; 95% CI [24.9%; 27.3%]). Men were more commonly affected than women (tinnitus prevalence of 30.2% in men (*n* = 770) and 21.7% in women (*n* = 519), *p*-value < 0.0001). Tinnitus prevalence increased with age, peaking at 31.2% in the 75–79 age group (Figure 2). Only 6.7% of the participants who reported having tinnitus also reported having been treated for their tinnitus in the past two years.

Tinnitus prevalence increased with increasing hearing impairment according to WHO classification, with a peak prevalence of 78.6% (11/14) among those with severe hearing impairment. Only one participant was tested for profound hearing impairment and reported no tinnitus. Seven participants had complete hearing impairment and three of them reported having tinnitus (42.9%). This effect was statistically significant (Table 2). Of the participants with tinnitus, 39.7% (504/1271) may have needed a hearing aid based on pure-tone audiometry, compared to 26.9% (979/3637) of participants without tinnitus (*p*-value < 0.0001).

**Figure 3:**
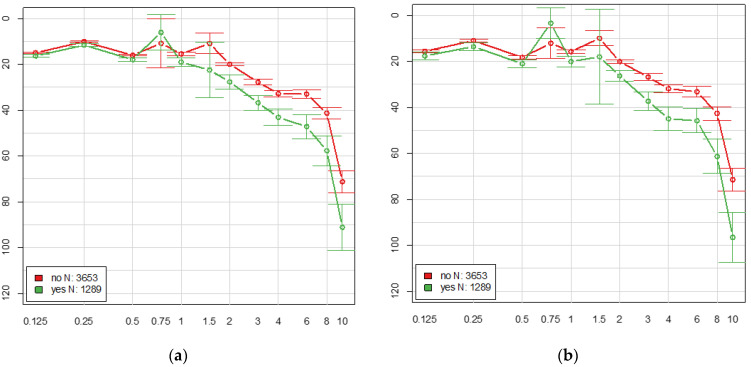
Mean hearing levels (pure-tone audiometry, air conduction), *x*-axis: frequencies in kHz, *y*-axis: mean hearing level in dB. (**a**) left ear, (**b**) right ear; red: participants without tinnitus, green: participants with tinnitus.

Participants with tinnitus showed significantly higher mean hearing levels (air conduction) than participants without tinnitus at frequencies of 4 kHz or higher (Figure 3). The mean differences at 4/6/8/10 kHz were 9/15/10/5 dB for the left ear and 10/10/15/10 dB for the right ear (*p*-value < 0.0001).

**Figure 4:**
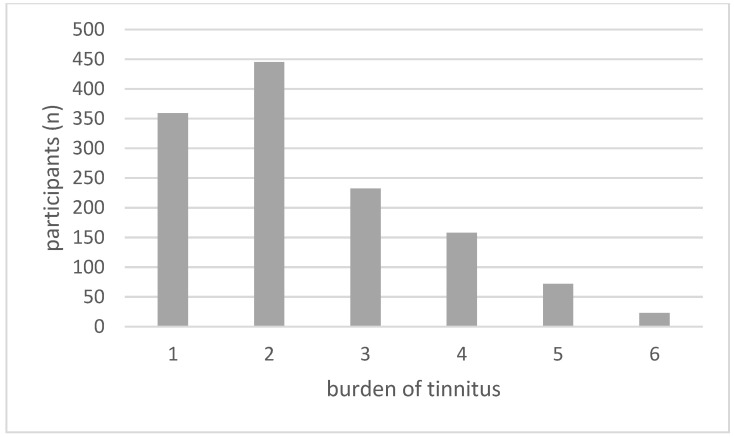
Burden of tinnitus among participants with tinnitus (1 = “little stressful” to 6 = “extremely stressful”).

On a scale from 1 to 6, with 1 representing “little stressful” and 6 representing “extremely stressful”, the mean reported burden among participants with tinnitus was 2.39 (standard deviation (SD): 1.26) for the entire cohort (Figure 4).

Women were significantly more burdened by their tinnitus (women: 2.51 (SD: 1.31), men: 2.30 (SD: 1.22), *p*-value = 0.0039). Age had no statistically significant effect on perceived burden of tinnitus (*p*-value: 0.35) when sex and hearing impairment were taken into account. Participants with severe hearing impairment felt most bothered by their tinnitus (3.45 (SD: 1.81); *p*-value < 0.0001) as compared to other participants with tinnitus with either no hearing impairment (2.22 (SD:1.13) or hearing impairment other than a severe impairment (mild: 2.47 (SD: 1.31); moderate: 2.70 (SD: 1.42); moderately severe: 2.55 (SD: 1.36); complete: 1.33 (SD: 0.58)). With a tinnitus burden of 3–6 as an annoying tinnitus, the prevalence of annoying tinnitus was 9.8% (485/4942) in the total cohort (10.8% in men (275/2550) and 8.8% in women (210/2392)). 

Logistic regression results showed that participants with severe to complete hearing loss (>65 dB) were more likely to have tinnitus (OR = 6.53, 97.5% CI [2.57; 16.59], *p*-value < 0.001) than participants without hearing loss. Sex and mild to moderately severe hearing loss also had a significant effect on tinnitus, while age did not influence its occurrence (Table 3).

Generalizing the prevalence results weighted to the ESP 2013, the overall prevalence of tinnitus is 26.2% (men: 30.5%, women: 21.9%). When only age groups below 85 years are considered, the overall prevalence of tinnitus is 26.2% (men: 30.5%, women: 21.8%).

## 4. Discussion

The aim of this study is to determine the prevalence of tinnitus based on a large, population-based cohort. Due to its design, the results are representative for the region of Mainz-Bingen and can be generalized to the European population. In addition to the prevalence results, this study adds to the literature by describing the association between tinnitus and hearing loss from an epidemiological perspective.

Tinnitus is a common problem. We found that one in four people in our cohort suffers from it. In general, men are affected more often than women. As hearing loss progresses, the prevalence of tinnitus increases. Despite the high prevalence of tinnitus, only a minority of patients seem to consult a doctor. Considering a burden of 3–6 (on a six-level scale) as an annoying tinnitus, the prevalence in our cohort was 9.8%.

Reports of tinnitus prevalence have been published in the literature describing a wide range from 5.2% to 45% of tinnitus prevalence [4,7,8,10,23,24,25,31,32,33,34,35,36,37,38,39,40,41,42,43]. However, most studies describe a prevalence between 10 and 15% [1]. This diversity is mainly due to different definitions of tinnitus (e.g., lasting longer than 5 min, or perceived as bothersome). Furthermore, tinnitus prevalence depends on the age structure of the population studied. In European cohort studies with a similar age structure as our cohort, a prevalence between 17 to 29.2% was found [4,36,43]. Our result of 26.1% is in line with these results. When tinnitus and hearing loss have been associated, hearing information has often been based on self-reporting in an interview or mailed questionnaire [7,25,33,38,39,40]. However, self-reporting may not be sufficient to detect hearing loss [15]. Most studies have found an increase in tinnitus prevalence with age and a higher rate in men [7,8,10,23,24,33,34,35,36,41]. In addition, the few studies that have used pure-tone audiometry to determine the hearing threshold have found that hearing impairment is associated with the occurrence of tinnitus [10,41,42]. This is consistent with our study.

A recent report on a population-based cohort from the Netherlands found a tinnitus prevalence of 21.4%, which is slightly lower than our study result. The cohort’s average age was 69.4 years and therewith slightly older than our age average. They also reported a higher prevalence in males. Unlike our study, they found no overall variance in tinnitus prevalence between different age groups. However, their finding that hearing impairment increases the likelihood of tinnitus is in line with our results [44].

Although many studies on tinnitus prevalence are based on a European cohort, these studies are mainly from the United Kingdom, the Netherlands and Scandinavia [44,45]. The only comprehensive analysis in Germany was conducted in the year 1999 [46]. It surveyed 3000 residents in Germany by telephone and described a tinnitus prevalence of 3.9%.

Overall, this study adds to the literature by reporting tinnitus prevalence in a large, population-based cohort. Participants were interviewed and tested for hearing impairment using pure-tone audiometry. With this in mind, this is the first study known to the authors to provide a comprehensive report on the prevalence of tinnitus and its association with hearing loss in Germany. The recent updates to the tinnitus guidelines show that tinnitus remains a topic of interest, and given the many innovations in tinnitus research, we believe that providing current prevalence figures is important to understand the occurrence of tinnitus in a changing society [47].

This study contains limitations. First, as discussed by McCormack et al. in their review, there is no standard definition of tinnitus for research purposes [45]. This makes comparison between different studies difficult. By asking “Do you suffer from ringing in the ears (tinnitus)?” our assessment was relatively broad compared with studies that asked only about annoying tinnitus. This will be the most important reason for the relatively high prevalence compared with other studies. To reduce this limitation, we also report the prevalence of annoying tinnitus, although the cutoff of 3 on a six-level scale can be criticized as somewhat arbitrary. In addition, we cannot further differentiate in which dimension or to which extent the tinnitus burden reported by the participants influences their quality of life or whether it affects their psychosocial well-being. Tinnitus certainly interacts with many psychological comorbidities. Reports of this association can be found elsewhere and therefore where not the subject of this study [48].

In addition, we included age, sex, and hearing impairment as possible risk factors for the occurrence of tinnitus. Other factors such as stress or comorbidities (e.g., depression) were not part of this analysis. This needs to be further analyzed in future studies.

## 5. Conclusions

Tinnitus is a common condition. As it increases with age, we must assume a rising prevalence in the ageing population. However, only a minority of those affected are significantly distressed by their tinnitus. It will be important for the practicing physician to identify these patients.

## Figures and Tables

**Table 1:** Descriptive statistics and main results.

	All	Men	Women	*p*-Value
*n*	4942	2550	2392	
age, mean (SD)	61.0 (13.3)	61.8 (13.4)	60.2 (13.2)	<0.0001 *
tinnitus (yes)	26.1%	30.2%	21.7%	<0.0001 *
burden due to tinnitus, mean (SD)	2.39 (1.26)	2.30 (1.22)	2.51 (1.31)	0.0039 *
annoying tinnitus (burden of 3–6)	9.8%	10.8%	8.8%	0.0233 *

* Values represent significance with *p* < 0.05.

**Table 2:** Tinnitus prevalence depending on hearing loss according to WHO grading of hearing impairment.

WHO Hearing Impairment	Audiometric Value (Average across 0.5/1/2/4 kHz, Better Ear)	Tinnitus Prevalence [95% CI]
No impairment	<20 dB	21.6% [20.1%;23.1%]
Mild impairment	20–34.9 dB	31.2% [28.8%;33.8%]
Moderate impairment	35–49.9 dB	34.2% [30.0%;38.7%]
Moderately severe impairment	50–64.9 dB	38.5% [29.2%;48.5%]
Severe impairment	65–79.9 dB	78.6% [48.8%;94.3%]
Profound impairment	80–94.9 dB	0%
Complete impairment	≥95 dB	42.9% [11.8%;79.8%]

CI = confidence interval.

**Table 3:** Logistic regression of tinnitus on sex, age and hearing impairment (by WHO classification).

Variable	OR	2.5%	97.5%	*p*	Events	*n*	z-Score	Nagelkerke R^2^
					1289	4941		0.036
Sex (female)	0.67	0.59	0.77	<0.001	519	2392	−5.986	
Age (per year)	1.00	1.00	1.01	0.55			0.545	
Mild to moderately severe hearing impairment *	1.63	1.39	1.39	<0.001	641	1981	5.963	
Severe to complete hearing impairment *	6.09	2.52	14.72	<0.001	14	22	4.011	

* By WHO classification; z-score for intercept: −6.759.

## Data Availability

Not applicable.
